# Medical Items

**Published:** 1895-02

**Authors:** 


### *MEDICAL ITEMS.*

---

MAINE, New Hampshire, Vermont, Massachusetts (with a law which is no law), and Rhode Island are almost the only States now without restrictive medical laws. What a nice comment upon the boasted intelligence and culture of New England!

---

WE are told (by Dr. Kraft, Homeopathic *Clinical Reporter*) that sleeping with hands over the head often indicates (in women) a tendency to falling of the womb. What that symptom indicates in men, or what symptom indicates that condition in men, we are not informed.

---

WE beg to thank the publisher for "Landmarks in Gynecology," by Dr. Byron Robinson of Chicago. Two volumes. Also for "Where to Send Patients Abroad for Water Cures, and Climatic Treatment," by Dr. Thomas Linn of Paris. "Physicians' Leisure Library Series." Price, twenty-five cents per volume. Geo. S. Davis, Publisher, Detroit, Mich.

---

THE trial of Dr. A. B. Hinkle, of Americus, for the murder of Dr. Worsham, in 1893, resulted in an acquittal last month. It will be remembered that Dr. Hinkle's father was convicted of the same murder a year ago and sentenced to be hanged. He committed suicide in prison. The defence of the younger Hinkle was that the killing was done by his father.

---

MESSRS. FREDERICK STEARNS & CO., Detroit, have published an interesting monograph on the Kola nut, describing in a thoroughly scientific manner its physical and medicinal properties. It contains also an exhaustive compilation of all the literature relating to the Kola from 1591 to the present time. The monograph will be sent gratuitously to physicians applying for it.

---

WE have received the second issue of Maine *Journal of Medicine and Science* (Portland), the official organ of the Maine Academy of Medicine. The *Journal* announces a "circulation of 30,000." Considering that Maine has only 1,200 physicians, we should say that the new journal begins life under the most brilliant prospects, and that the "organ" is in a very healthy condition.

---

AN obituary in a late Georgia newspaper contained the following:

"I gave him his medicine regular,
From morn to the set of sun.
He took two powders at 10 o'clock,
And another powder at one.

"But doctors cannot help us,
When death knocks at the door;
Goodby, my darling husband!
You left at 10 minutes to 4!"

---

Now it is stated (Dr. C. H. Lindsley, Conn.) that some cases of typhoid fever among the students of Wesleyan University were lately traced to oysters "grown near the mouth of a sewer connecting with a house in which there were two cases of typhoid. The typhoid germs had not lost their virulence by entering the circulation of the oyster." We reprint this interesting story as a companion to the one now going the rounds about bedbugs as carriers of tubercle bacilli. Such brilliant examples of *fin de siècle* medical detective work would put to shame the ingenuity of Mr. Sherlock Holmes in his palmiest days. *Hæc fabulæ docent:* Oysters and bedbugs should be thoroughly sterilized before using.

MESSRS. WILLIAM WOOD & COMPANY, New York, have begun to publish the largest and most important medical work of the century, to be entitled the "Twentieth Century of Practice." It is intended to be a "fine presentation, by master-hands, of medicine as it is at the close of the nineteenth and beginning of the twentieth century." It will be published in twenty volumes, one volume about every three months. The series will therefore be completed by the end of 1899. A long list of able contributors have been carefully selected. It will be an achievement in *fin de siècle* medical publication, and will merit a cordial reception by the profession.

WE regret to give notice of the death of Dr. Alfred L. Loomis, of New York, which occurred January 23d, from acute pneumonia. For many years Dr. Loomis has been prominent in American medicine, both as teacher and author, and his medical writings have been popular and successful. The best known of these are his "Text-Book of Practical Medicine," which has reached six editions, and his "Lessons in Physical Diagnosis," ten editions.

He was also the author of many valuable monographs relating to the diseases of the heart, lungs, kidneys, etc. Dr. Loomis was visiting physician to Bellevue Hospital for thirty years, and since 1868 had been professor of pathology and practice of medicine in the University of the City of New York. Dr. Loomis was in his sixty-fourth year.

---

THE Meriwether County Medical Society held its first meeting in Greenville on Saturday, January 5th. The names enrolled for membership were: Drs. E. B. Terrell of Greenville; H. W. Terrell of Greenville; W. H. Goodwin of Bullochville; T. J. Hatchett of Raleigh; E. C. Thrash of Gay; J. W. Taylor of Lutherville; Jno. W. Taylor of Lutherville; A. Q. Young of Lutherville; H. J. Lasseter of Lutherville.

The meeting was then permanently organized by electing Drs. H. W. Terrell, President, E. C. Thrash, Secretary, and A. Q. Young, Treasurer. Drs. H. W. Terrell, H. W. Clements, and A. Q. Young were appointed to draft a constitution and by-laws.

---

SAY! This may mean you. Every periodical now and then gets one of its copies back with "refused" marked on the cover. We think Bill Nye was about right when he said: "A man may ride on the back coach of a railroad train to save interest on his money till the conductor gets around, or stop his watch at night to save wear and tear, or leave his 'i' or 't' without a dot or cross to save ink, or pasture his mother's grave to save corn, but a man of this sort is a gentleman compared to the fellow that will take a paper two or three years, and when asked to pay for it puts it back in the office and has it marked 'refused.'" The above is to whom it may concern, or may be likely to concern at any time.

